# A Comparative Elemental and Surface Analysis of Root Cementum in Severe Periodontitis and Healthy Teeth

**DOI:** 10.1055/s-0045-1806959

**Published:** 2025-05-07

**Authors:** Sahar M. N. Bukhary, Hisham I. Othman, Ghada Mansour, Madawi F. Alkeheli

**Affiliations:** 1 Department of Oral Biology, Faculty of Dentistry, King Abdulaziz University, Jeddah, Saudi Arabia; 2 Department of Oral Diagnostic Sciences, Faculty of Dentistry, King Abdulaziz University, Jeddah, Saudi Arabia; 3 Department of Oral Biology, Faculty of Dentistry, Alexandria University, Alexandria, Egypt; 4 Department of Oral Medicine, Periodontology, Oral Diagnosis and Oral Radiology, Faculty of Dentistry, Alexandria University, Alexandria, Egypt

**Keywords:** elemental analysis, periodontitis, cementum surface, EDX, SEM

## Abstract

**Objective:**

This study aims to compare the elemental composition and surface characteristics of root cementum in teeth affected by severe periodontitis with those of healthy teeth.

**Materials and Methods:**

Forty-seven teeth, including 25 teeth affected by stage III, grade C periodontitis and 22 healthy teeth, were extracted from patients aged 17 to 34 years. The cementum surfaces were analyzed using scanning electron microscopy (SEM) and energy-dispersive X-ray spectroscopy (EDX) to evaluate surface morphology and elemental composition.

**Results:**

SEM images revealed that healthy teeth exhibited a homogenous, smooth cementum surface, while teeth affected by periodontitis showed an irregular, uneven surface with deep crack lines and resorption areas. EDX analysis indicated significant differences in elemental composition; periodontitis-affected teeth had lower calcium and phosphorus but higher magnesium, sodium, and sulfur levels than healthy teeth.

**Conclusion:**

Periodontitis significantly alters the surface characteristics and elemental composition of root cementum, which may contribute to disease progression and impaired periodontal health.

## Introduction

Cementum, due to its intermediary position between root dentin and periodontal ligament (PDL), is a component of the tooth and belongs functionally to the periodontium. Periodontitis is characterized by microbially associated, host-mediated inflammation that results in loss of periodontal attachment. Moreover, genetics, environmental, and behavioral factors are involved in its development and speed of progression. Certain demographic characteristics, such as age, gender, ethnicity, and socioeconomic status, influence the prevalence of periodontitis. Others strongly contributing factors include smoking, diabetes mellitus, metabolic syndrome, and obesity. 1 2 The progression of periodontal disease results in the loss of periodontal attachment from the root surface and the exposure of cementum to the oral environment. Periodontal disease significantly impacts the root surface, leading to various alterations, including hypermineralization of the cementum surface, degradation of the collagen matrix, and the formation of resorption lacunae. 1 The presence of bacterial endotoxins in the exposed cementum plays a crucial role in this process, as they penetrate the surface and contribute to these structural modifications. 2 These changes highlight the complex interplay between periodontal pathogens and the host response, influenced by multiple environmental and genetic factors, elucidating the underlying mechanisms involved in the progression of periodontal disease. 3

In 2018, a new classification of periodontal and peri-implant diseases and conditions was introduced by a joint World Workshop composed of the American Academy of Periodontology (AAP) and the European Federation of Periodontology (EFP). 4 This classification focuses on staging of the disease (stages I–IV) based on severity, extent, and complexity of management, and disease grading (grades A–C) according to rate of progression and response to treatment. Staging is determined after considering variables including clinical attachment loss, amount and percentage of bone loss, probing depth (PD), presence and extent of angular bony defects and furcation involvement, tooth mobility, and tooth loss due to periodontitis. Grading includes three levels (grade A—low risk, grade B—moderate risk, and grade C—high risk for progression) and encompasses, in addition to aspects related to periodontitis progression, general health status, and other exposures such as smoking or level of metabolic control in diabetes. 5

Stage III, grade C periodontitis, describing severe periodontal breakdown with high risk of progression, is characterized by rapid attachment loss and bone destruction, and a lack of consistency between clinically visible bacterial deposits and the severity of periodontal breakdown. 5 Various causes have been previously proposed for severe periodontitis, including immunodeficiency in patients, bacterial invasion, genetic factors, and defective cementogenesis of the affected teeth. These insights suggest a multifactorial etiology requiring comprehensive diagnostic and therapeutic approaches. 6

Energy-dispersive X-ray spectroscopy (EDX), when combined with scanning electron microscopy (SEM), provides a powerful tool for detailed chemical and elemental analysis. EDX works by scattering the X-ray spectrum with sufficient sensitivity to display comprehensive X-ray spectral data. The technique's effectiveness hinges on the principle that each element has a unique atomic structure, resulting in a distinct set of peaks in its X-ray emission spectrum. This allows for precise identification and quantification of elements within the sample. EDX is particularly valuable for its ability to provide full quantitative analysis of sample composition, making it indispensable in the fields ranging from materials science to biology. Recent advancements in EDX technology have further enhanced its resolution and accuracy, allowing for even more detailed investigations at the micro- and nanoscales. 7

Periodontal disease impact on the root surface has been previously evaluated. In periodontitis, chemical analysis of exposed cementum revealed marked variation in mineral elemental composition. Such alterations in mineral composition have significant implications for the structural integrity and biological function of the cementum, potentially affecting periodontal attachment and disease progression. This understanding underscores the necessity for further research into the pathophysiological mechanisms underlying these mineral changes and their impact on oral health. 8 Conversely, another research has indicated that in the cementum of exposed root surfaces, there were no significant differences in calcium (Ca) and phosphorus (P) contents. 9 These discrepancies have been attributed to the different preparative methods used in various studies, which may alter the chemical composition of the root surface, as well as the heterogenicity of the studied groups with variable degrees of periodontitis severity. Additionally, advanced imaging techniques have highlighted the variability in mineral distribution within the cementum, emphasizing the need for standardized protocols in future research. 10

## Objective

Given the critical role of cementum in periodontal attachment, this study aims to compare the elemental composition and surface characteristics of root cementum in teeth affected by stage III, grade C periodontitis (severe periodontitis with a high risk of progression) with those of healthy teeth. Understanding these differences may provide insights into the pathogenesis of the disease and aid in developing more effective therapeutic strategies, such as biological mediators, stem cell therapy, and advanced biomaterials.

## Materials and Methods

The study was approved by the research ethical committee of our institution (no. 134-11-24), and informed consent was obtained from all participants.

This study involved 47 teeth extracted from patients at our institute. Participants were generally healthy, nonsmokers and had not received antibiotics or periodontal therapy in the past 6 months. Their age ranged from 17 to 34 years.

Extracted teeth were distributed in two groups as follows:

*Group I (control)* : This group consisted of 22 periodontally healthy, sound teeth that required extraction for orthodontic reasons. They were age-matched with periodontitis patients in group II. These teeth exhibited neither destruction of gingival attachment nor bone loss, ensuring a baseline for healthy periodontal conditions.
*Group II (periodontitis)* : This group comprised 25 periodontally diseased teeth collected from 14 patients diagnosed with stage III grade C periodontitis (severe periodontitis with high risk of progression), as diagnosed by a periodontist. The diagnosis was based on the clinical and radiographic criteria established by the AAP and the EFP. 4 5 A full-mouth series of periapical radiographs was performed. Before extraction, percentage of bone loss from radiographs, PD, and clinical attachment level (CAL) were recorded.

### Criteria of Selection

*Stage III* : Interproximal CAL >5 mm, radiographic bone loss: extending to middle third of the root and beyond, tooth loss due to periodontitis of <4 teeth, PD >6 mm, vertical bone loss >3 mm, furcation involvement class II or III.
*Grade C* : Specific clinical picture suggestive of rapid progression and/or early-onset disease (e.g., molar/incisor pattern, lack of expected response to standard bacterial control therapies), % bone loss/age: >1, destruction not consistent with visible bacterial deposits.

Patients were excluded from the study if they met any of the following exclusion criteria: systemic diseases that might have affected the thickness of cementum, parafunctional habits, localized periapical pathology, reimplanted teeth, any kind of pulpal conditions affecting root surfaces, radiographic evidence of hypercementosis or root resorption, trauma from occlusion, teeth without antagonists, unilateral chewing habit, open bite, and periodontal treatment (mechanical or chemical) within the past 6 months.

Before extraction, the eligible patients were requested to leave their teeth voluntarily after an explanation of the purpose of the study.

The teeth were collected and fixed in 2.5% buffered glutaraldehyde to preserve the tissue morphology and prevent degradation. Cross-root sections were cut at the cementoenamel junction to ensure a consistent and relevant anatomical reference point for analysis. The selected root surface areas were determined for detailed examination using a (SEM) and an energy-dispersive X-ray analyzer (EDX) unit, allowing for high-resolution imaging and elemental analysis of the root surfaces. These methods provided comprehensive insights into the surface characteristics and compositional elements of the dental samples, critical for understanding the differences between healthy and diseased teeth. 11

### Statistical Analysis

The collected data were statistically evaluated using Student's *t* -test to determine the significant differences between the groups. The level of significance was set at *p* < 0.001.

## Results

### Surface Characteristics

**Fig. 1:**
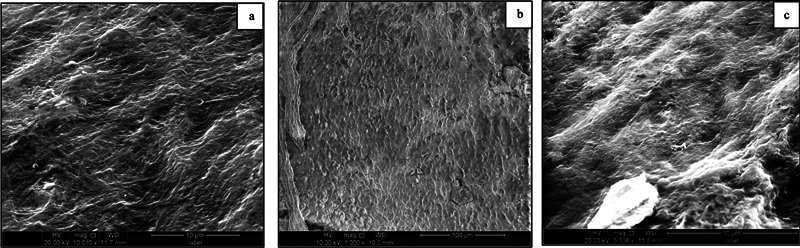
Scanning electron micrograph of group I (control) at the (a) cervical, (b) middle, and (c) apical thirds of the root showing a uniform smooth cementum surface with numerous projections of the periodontal fibers.

SEM images showed that the cementum surface of sound teeth (Group I) displayed a homogenous, regular, and smooth appearance, with periodontal fibers tightly embracing the cementum ( Fig. 1 ).

**Fig. 2:**
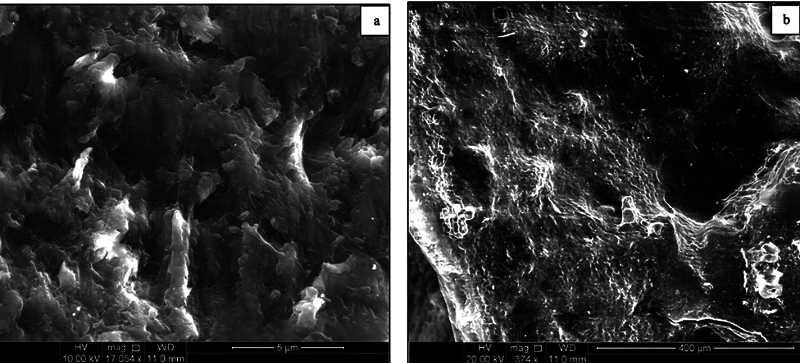
Scanning electron micrograph of group II (severe periodontitis) at the (a) cervical third of the root showing an irregular, rough surface with multiple defect areas in the cementum surface. (b) Multiple deep defect areas of variable depths.

**Fig. 3:**
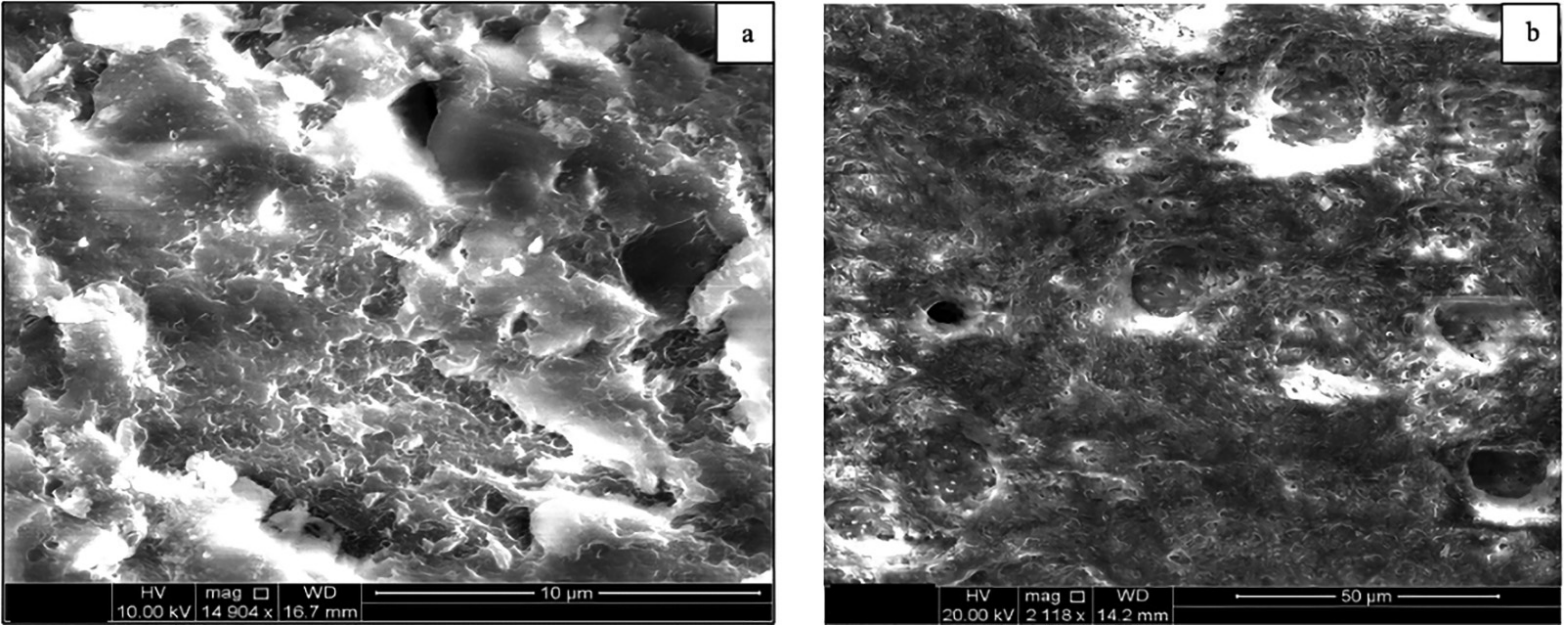
Scanning electron micrograph of group II (severe periodontitis) at the (a) middle third of the root showing multiple defect areas scattered on the destructed cementum surface with the absence of the periodontal fibers. (b) Deep defect cavities exposing the underlying dentinal tubules.

**Fig. 4:**
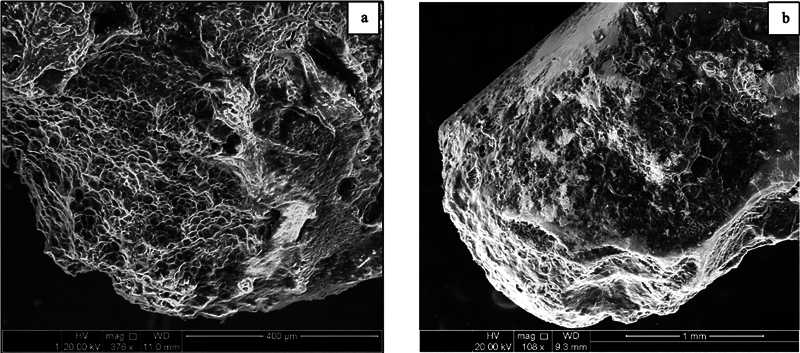
Scanning electron micrograph of group II (severe periodontitis) at the (a) apical third of the root showing multiple areas of hypoplasia scattered on the root surface with the absence of the covering cementum. (b) Deep defect cavities expose the underlying dentinal tubules with extensive cementum degradation.

In contrast, the cementum of advanced periodontitis teeth (Group II) exhibited an irregular and uneven surface, marked by multiple defect areas of varying sizes and depths at the cervical ( Fig. 2 ) and middle thirds of the roots ( Fig. 3 ). Additionally, deep crack lines were widely distributed across the entire cementum surface, indicating significant structural compromise. There was a complete absence of periodontal fibers, and numerous resorption areas extended deep into the underlying dentin at the apical third of the root ( Fig. 4 ).

### Elemental Composition

EDX analysis revealed significant differences between the control and the periodontitis groups in the concentrations of key elements, specifically levels of Ca, P, S, Na and Mg.

**Fig. 5:**
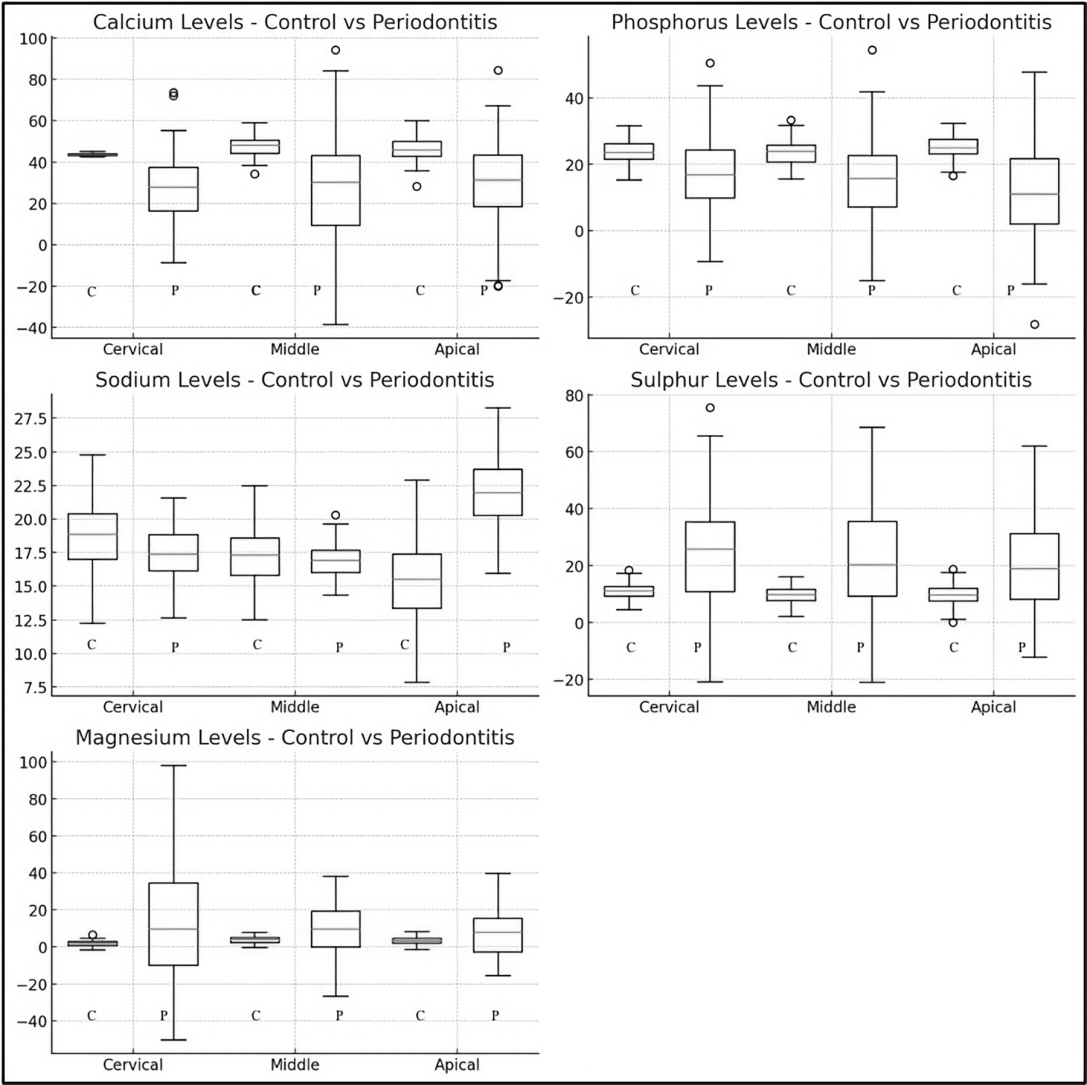
Box plots with whiskers show higher levels of calcium and phosphorus, and lower variability in magnesium in the control group. The periodontitis group, on the other hand, demonstrates greater variability and higher levels of magnesium and sulfur in certain regions. (C = control group, P = periodontitis group).

The control group showed significantly higher concentrations of Ca and P across all root regions compared with the periodontitis group. Conversely, the periodontitis group exhibited significantly higher concentrations of Mg, sodium (Na), and S across all root regions compared with the control group ( Table 1 , Fig. 5 ).

**Table 1:** Statistically significant differences between the control and periodontitis groups across most minerals, with the control group generally showing higher levels of calcium and phosphorus, while the periodontitis group exhibits elevated levels of sulfur and magnesium

Element	Region	Control group, mean ( *n* = 22)	Periodontitis group, mean ( *n* = 25)	Mean differences	SD	*p* -Value
Calcium	Cervical	41.75	26.36	17.39	0.5	<0.0001
	Middle	47.5	25.33	22.17	4.5	<0.0001
	Apical	46.25	27.22	19.03	4.7	<0.0001
	Summation	137.5	78.92	58.58	9	<0.0001
Phosphorus	Cervical	24.25	15.89	8.36	3.5	<0.0001
	Middle	23	14.36	8.64	3.2	<0.0001
	Apical	25	13.67	11.33	3.6	<0.0001
	Summation	72.25	43.92	28.33	7	<0.0001
Sodium	Cervical	18.63	17.37	1.26	2.5	0.0519
	Middle	17.5	16.97	0.53	2.2	0.2345
	Apical	15.5	22.06	−6.56	2.8	<0.0001
	Summation	51.63	56.4	−4.77	5	<0.0001
Sulfur	Cervical	11	23.69	−12.69	3	<0.0001
	Middle	9.5	22.11	−12.61	2.8	<0.0001
	Apical	9.89	20.1	−10.21	3.2	<0.0001
	Summation	30.39	65.9	−35.51	6.5	<0.0001
Magnesium	Cervical	1.75	9.71	−7.96	1.5	<0.0001
	Middle	3.88	8.14	−4.26	1.8	<0.0001
	Apical	3.17	9	−5.83	2	<0.0001
	Summation	8.79	26.85	−18.06	4.5	<0.0001

Abbreviation: SD, standard deviation.

Note: These findings suggest that periodontitis may significantly impact the mineral composition in dental tissues.

In box plots with whiskers diagrams ( Fig. 5 ), the Ca and P consistently show higher concentrations across all regions in the control group compared with the periodontitis group. The distributions in the control group are tighter, indicating less variability, in contrast to the periodontitis group that exhibits greater variability in these elements.

## Discussion

In the current study, the cementum surface in teeth affected by periodontitis exhibited multiple areas of hypoplasia. These hypoplastic areas were observed on all examined teeth and across all root surfaces. Such findings may be attributed to the vulnerability of the cementum of periodontally affected teeth to the oral environment. Our results align with previous similar findings of cementum hypoplasia in teeth affected by severe periodontitis. 8 These changes underscore the significant impact of periodontal disease on the structural integrity of dental tissues and the extensive cementum degradation associated with severe periodontitis, highlighting the severe disruption of periodontal architecture.

Earlier studies have further validated the hypothesis that abnormal defective cementum significantly enhances pathogen invasion and extensive bone loss in periodontal disease. It has been demonstrated that the structural integrity and biochemical composition of the cementum matrix are severely compromised in periodontal disease, with the provisional matrix produced during periodontal regeneration differing markedly from that of normal cementum. During periodontal healing, a battery of growth factors is available from both the circulatory and inflammatory cells, and the provisional matrix is not likely to be conducive to the function of cementoblast progenitors, resulting in the unavailability of differentiation of cementoblasts, as cementogenesis signaling molecules are not readily available in the wound healing environment. 8 12 Moreover, evidence indicates that defective cementum may predispose individuals to periodontal attachment loss and advanced periodontal destruction by increasing the periodontium's susceptibility to bacterial infection. 13 These findings have been further investigated and emphasized the critical role of cementum in maintaining periodontal health and the importance of targeting cementum integrity in periodontal therapies. 14 15 Furthermore, it has been demonstrated that in patients with severe periodontitis, cytokines and inflammatory mediators stimulate periodontal breakdown and collagen destruction via tissue-derived matrix metalloproteinases. 16 17

Moreover, in the present work, elemental analysis revealed a significant difference between the periodontitis and nonperiodontitis groups in terms of the mineral content along the three root thirds, with decreased Ca and P levels in the periodontitis group. In contrast, dissimilar findings were reported by others, who reported that diseased root surfaces have revealed higher levels of Ca and P compared with nondiseased surfaces. These researchers attributed their results to the exposure of the root surface to the oral environment, which facilitates mineral exchange at the cementum–saliva interface, resulting in a hypermineralized cementum surface. 18 In our study, the decrease in Ca and P levels is likely due to bone resorption and disrupted mineral metabolism, where Ca is mobilized from the bone into the bloodstream, reducing local Ca levels in the affected areas. 19 P works closely with Ca to form hydroxyapatite; inflammation in periodontitis can interfere with P metabolism, leading to decreased local P levels.

On the other hand, increases in Mg, Na, and S levels were detected in the present work. These findings may indicate an active inflammatory response and tissue degradation. Similar findings were reported by other investigators. 20 These changes reflect both the destructive processes and the body's attempts to repair and respond to ongoing inflammation. 21 The variation in mineral content of periodontally involved roots can be attributed to the exposure of the root to saliva and the surrounding infected environment through gingival recession, bone loss, and pocket formation. 1 Moreover, such changes in the mineral composition are consistent with the pathological processes of periodontitis, where the breakdown of periodontal structures leads to altered mineralization patterns. 22

The alteration in cementum structures and composition due to periodontal disease has significant implications for periodontal therapy. An essential objective of periodontal regeneration is the restoration of the structural and functional integrity of the periodontium, including alveolar bone, PDL, new cementum, and the re-establishment of connective tissues and epithelial adhesion to the cementum. It has been reported that the integrity of cementum is compromised by periodontal disease, highlighting the pivotal role of the effect of this alteration on periodontal regeneration. 23 Therapeutic approaches for periodontitis should focus on creating a cementum microenvironment that promotes new cementum formation as part of periodontal regeneration. Current methods to support this objective include root conditioning, the application of growth factors, enamel proteins, and barrier membranes. However, these methods have remarkable limitations. For instance, root conditioning exposes molecules such as type-I collagen, which has poor cell specificity and does not re-establish the unique composition of the cementum microenvironment. 24 Similarly, barrier membranes, while facilitating the population of the treated site by desired cells, do not restore the unique composition of the cementum local environment necessary for cellular differentiation. 25 Enamel matrix proteins may assist in early cementogenesis but lack the ability to recruit and differentiate cementoblast progenitors in adults. 26

Moreover, recent regenerative strategies have been investigated for preclinical and clinical situations and developed for periodontal tissues, such as implantable scaffolds or biologic delivery systems. 27 Major approaches in periodontal tissue engineering have focused on the development of bone substitutes or alveolar bone regeneration materials; however, the regeneration and configuration of PDL–cementum complexes still depend on proliferation or differentiation in PDL–cementum interfaces for periodontal revitalization, following histophysiological adaptations of the periosteum. 28 Biomaterial-based periodontal tissue engineering three-dimensional printing techniques have been employed to develop various scaffolding designs for periodontal tissue regeneration to compartmentalize individual tissues such as cementum, PDL, and the alveolar bone. These novel techniques will be the principal and predominant strategies for the new paradigm of periodontal regenerative medicine with greater predictability and high controllability. 29

The small sample size is the main limitation of this study. Further studies may be needed to investigate the elemental and surface analysis of the root cementum in other stages and grades of periodontitis, with less periodontal destruction to better understand if these changes are correlated with the disease severity.

## Conclusion

This study highlights significant differences in the root cementum of teeth affected by severe periodontitis with a high risk of progression compared with healthy teeth. These alterations in elemental composition and surface characteristics may contribute to the disease pathogenesis and progression. Further research is needed to explore the potential for targeted therapies aimed at restoring the integrity of root cementum in periodontal disease.
